# Evaluation of specific humoral immune response in pigs vaccinated with cell culture adapted classical swine fever vaccine

**DOI:** 10.14202/vetworld.2016.308-312

**Published:** 2016-03-25

**Authors:** Mrinal K. Nath, D. K. Sarma, B. C. Das, P. Deka, D. Kalita, J. B. Dutta, G. Mahato, S. Sarma, P. Roychoudhury

**Affiliations:** 1 Department of Veterinary Epidemiology and Preventive Medicine, College of Veterinary Science, Assam Agricultural University, Khanapara, Guwahati, Assam, India; 2 ICAR-National Research Centre on Pig, Rani, Guwahati, Assam, India; 3 Department of Veterinary Microbiology, College of Veterinary Science, Assam Agricultural University, Khanapara, Guwahati, Assam, India; 4 ICAR - All India Co-ordinated Research Project on Pigs, College of Veterinary Science, Assam Agricultural University, Khanapara, Guwahati, Assam, India; 5 Department of Veterinary Bio-chemistry, College of Veterinary Science, Assam Agricultural University, Khanapara, Guwahati, Assam, India; 6 Department of Veterinary Microbiology, College of Veterinary Science and Animal Husbandry, Central Agricultural University, Aizawal, Mizoram, India

**Keywords:** antibody titer, classical swine fever vaccine, liquid phase blocking-enzyme-linked immunosorbent assay, pig

## Abstract

**Aim:**

To determine an efficient vaccination schedule on the basis of the humoral immune response of cell culture adapted live classical swine fever virus (CSFV) vaccinated pigs and maternally derived antibody (MDA) in piglets of vaccinated sows.

**Materials and Methods:**

A cell culture adapted live CSFV vaccine was subjected to different vaccination schedule in the present study. Serum samples were collected before vaccination (day 0) and 7, 14, 28, 42, 56, 180, 194, 208, 270, 284 and 298 days after vaccination and were analyzed by liquid phase blocking enzyme-linked immunosorbent assay. Moreover, MDA titre was detected in the serum of piglets at 21 and 42 days of age after farrowing of the vaccinated sows.

**Results:**

On 28 days after vaccination, serum samples of 83.33% vaccinated pigs showed the desirable level of antibody titer (log_10_ 1.50 at 1:32 dilution), whereas 100% animals showed log_10_ 1.50 at 1:32 dilution after 42 days of vaccination. Animals received a booster dose at 28 and 180 days post vaccination showed stable high-level antibody titre till the end of the study period. Further, piglets born from pigs vaccinated 1 month after conception showed the desirable level of MDA up to 42 days of age.

**Conclusion:**

CSF causes major losses in pig industry. Lapinised vaccines against CSFV are used routinely in endemic countries. In the present study, a cell culture adapted live attenuated vaccine has been evaluated. Based on the level of humoral immune response of vaccinated pigs and MDA titer in piglets born from immunized sows, it may be concluded that the more effective vaccination schedule for prevention of CSF is primary vaccination at 2 months of age followed by booster vaccination at 28 and 180 days post primary vaccination and at 1 month of gestation.

## Introduction

Classical swine fever (CSF) is a highly contagious viral disease of domestic and wild swine caused by genus Pestivirus of family *Flaviviridae*. The disease is considered as a major factor of economic losses to the swine industry and pig farming community [1-3].

Although E2 subunit marker vaccines have been developed in different countries for control of CSF [4], lapinized vaccines are still being used in many countries including India [5]. However, many outbreaks of the disease in vaccinated pig herd have been reported in India including North Eastern region [6]. Besides, lapinized vaccine doses being produced in India is not sufficient to immunize even 1% of the total pig population of the country. Thus, cell culture system will be more realistic to produce adequate doses of classical swine fever vaccines for the development of the pig industry in India. Cell culture attenuated CSF vaccine is also safe and produce a good level of immunity similar to the freeze dried lapinized vaccine. Besides in cell culture system, it is easy to determine the virus concentration [7].

Therefore, the present study was proposed to evaluate the kinetics of humoral immune response in cell culture adapted CSF vaccinated pigs as well as maternally derived antibody (MDA) of their offspring using different vaccination schedule.

## Materials and Methods

### Ethical approval

Ethical approval for the study was obtained from Institutional Animal ethics Committee of College of Veterinary Science, Assam Agricultural University, Khanapara.

### Vaccine

A live attenuated cell culture adapted vaccine (10^3^ tissue culture infective dose 50% per dose) developed by ICAR-National Fellow Project, Department of Microbiology, College of Veterinary Science, Assam Agricultural University, Khanapara, was used in the present study. The lapinised C strain of CSF virus (CSFV) was adapted in PK-15 cell line and after extensive field trials conducted by ICAR-National Fellow Project, the cell culture adapted C starin of the virus was found to be safe for immunization.

### Experimental animal

Twenty four CSF crossbred female piglets of 2 months old reared at ICAR - All India Coordinated Research Project on Pig, Assam Agricultural University, Khanapara were used for the present study. As per approval from the Institutional Animal Ethics Committee, all the animals were maintained under uniform dietary and managerial regime of the farm, and the experimental piglets were divided into four groups (Group A to D) comprising of six piglets in each group. Each group was kept isolated and supplied with separate feeding and watering troughs. Deworming of all the experimental animals were done prior to the commencement of the experiment. Further, before commencement of the experiment, serum samples of all the piglets were evaluated to ascertain that they are seronegative to CSFV and subjected to the following vaccination schedule by intramuscular route:

**Table:** 

Groups	Vaccination schedule						

	2^nd^ month of age	28 dpv	180 dpv	1 month of gestation
Group A	Primary vaccination	NV	1^st^ booster	NV
Group B	Primary vaccination	1^st^ booster	2^nd^ booster	NV
Group C	Primary vaccination	1^st^ booster	2^nd^ booster	3rd booster
Group D	Unvaccinated control group			

NV= Not vaccinated, dpv=Days post vaccination

### Serum samples and antibody detection

Serum samples were collected before vaccination (0 day) and 7, 14, 28, 42, 56, 180, 194, 208, 270, 284 and 298 days post vaccination (dpv) to monitor the humoral immune response of the experimental animals. In general, vaccination against CSF has routinely been performing at 6-month interval; therefore in the present study booster vaccination was attempted at 180 dpv. Further, to study the MDA titre, serum samples were collected from the piglets at 21 and 42 days of age born from vaccinated sows. To determine specific antibody level of the vaccinated herd as well as piglets born, in house liquid phase blocking (LPB) - enzyme-linked immunosorbent assay (ELISA) developed by the ICAR-National Fellow Project, Assam Agricultural University, Khanapara was performed according to the standard protocol with slight modification [8]. The percent inhibition in each well was calculated in relation to the respective antigen control using the formula given below, and the reciprocal of the serum dilution corresponding to 50% inhibition was considered as titer.


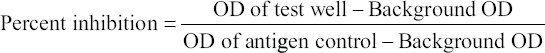


## Results

The percentages of animals showing LPB-ELISA antibody titre minimum of log_10_ 1.50 (1:32) against CSFV in different dpv are shown in Table-1. On the day of vaccination, none of the animals in any group showed antibody titre in serum samples. Out of six animals in each groups, one animal (16.67%) of group A and B and two animals (33.33%) of group C possessed log_10_ 1.50 (1:32) antibody titre on 14 dpv. Serum samples tested at 28 dpv revealed an increase in the numbers of animals showing such antibody titre (83.33% in all the groups). At 42 dpv, the antibody titre increased further and all animals in the vaccinated groups showed desired antibody level despite that animals of Group B and C received a booster dose on 28 dpv. All the animals within these groups were found to have maintained a stable antibody titre upto 56 dpv. Serum samples tested at 180 dpv revealed a decline in numbers of animals showing log_10_ 1.50 (1:32) antibody titre. None of the animals of group A showed the desired titre on 180 dpv but the numbers of animals possessing log_10_ 1.50 (1:32) antibody titre were five (83.33%) and four (66.67%) in Group B and C, respectively. Following revaccination on 180 dpv, the serum samples of 100% animals were found to possess desired LPB-ELISA antibody titre on 194, 208, 270, 284 and 298 dpv for all the groups. However, animals of Group C were again vaccinated during gestation after 1 month of conception (around 270 dpv). After farrowing piglets born from the sows of different Groups A, B, C and D were also tested by LPB-ELISA for detecting MDA at different periods of time (Table-2). Piglets born from all the sows of Group A, B and C were carrying the MDA to their offsprings. On 21 day of their age, one piglet in Group A (17.0%), five in group B (83.0%) and six numbers in Group C (100.0%) had a detectable level of MDA by LPB-ELISA. Further, none of the piglets in Group A, one piglet in Group B (17.0%) and four piglets in Group C (66.66%) were recorded to have MDA at 42 days of age. In the present study, none of the piglets of Group D showed MDA even at 21 days of their age.

**Table-1:** Percentage of pigs possessing LPB-ELISA antibody titre log_10_ 1.50 (1:32) against CSFV in different groups at different dpv.

Group	0 and 7 day	14 dpv	28 dpv	42 dpv	56 dpv	180 dpv	194 dpv	208 dpv	270 dpv	284 dpv	298 dpv
A	0.00	16.67	83.33	100.00	100.00	0.00	100.00	100.00	100.00	100.00	100.00
B	0.00	16.67	83.33	100.00	100.00	83.33	100.00	100.00	100.00	100.00	100.00
C	0.00	33.33	83.33	100.00	100.00	66.67	100.00	100.00	100.00	100.00	100.00
D	0.00	0.00	0.00	0.00	0.00	0.00	0.00	0.00	0.00	0.00	0.00

LPB-ELISA=Liquid phase blocking - enzyme-linked immunosorbent assay, CSFV=Classical swine fever virus, dpv=Days post vaccination

**Table-2:** Percentage of piglets born from immune sows possessing LPB-ELISA antibody titre log_10_ 1.50 (1:32) against CSFV in different groups at different age (days).

Groups	21 days	42 days
A	16.67	0.00
B	83.33	16.67
C	100.00	66.66
D	0.00	0.00

LPB-ELISA=Liquid phase blocking - enzyme-linked immunosorbent assay, CSFV=Classical swine fever virus

## Discussion

In India, pork meat production is the fastest growing segment of the livestock sector. However, CSF is endemic in India and causes huge losses to both the pork industry and backyard farmers. A study in 2011 study estimated the losses caused by CSF in India are around 2 billions INR [9]. Like many endemic countries, the control policy for CSF in India depends on vaccination against the disease to reduce or avoid serious economic losses.

**Figure-1:**
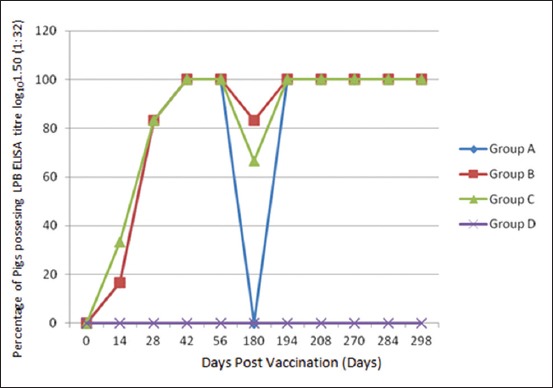
Percentage of pigs possessing liquid phase blocking - enzyme-linked immunosorbent assay antibody titre log_10_ 1.50 (1:32) against classical swine fever virus in different groups at different days post vaccination.

The internationally accepted methods for the detection of antibody response after vaccination are virus neutralization test (VNT), LPB-ELISA and solid phase competition ELISA [10]. In the present study, LPB-ELISA was standardized and used for antigenic characterization of CSFV isolates and compared the result with neutralization peroxidase linked assay (NPLA) and both the tests showed positive correlation [8]. On serological study of antibodies to foot and mouth disease virus, it is reported that 1:16 of VNT was equivalent to 1:32 by LPB-ELISA [11]. The LPB-ELISA is known to have a high correlation with serum neutralization test and could efficiently evaluate the potency of commercial vaccines [12, 13]. Thus, antibody titre log_10_ 1.50 (1:32) was considered as protective antibody titre to evaluate the potency of the presently used cell culture adapted CSF vaccine. All the animals included in the experiment were tested before vaccination and were found to be free from any detectable level of antibody titre in serum against CSFV. The result of LPB-ELISA revealed a gradual rise of antibody titre in all the three cell culture adapted CSFV vaccinated groups of animals (Figure-1). On 28 dpv, 83.33% animals of each vaccinated groups maintained the protective antibody titre and it further increased to 100% on 42 dpv and maintained this peak till 56 dpv. This pattern of protective antibody titre indicated that sufficient humoral antibodies conferred a stable protection to all animals which might be due to the high efficacy and a possibility of cell culture adapted CSF vaccine virus replication in host cells. Similar observation was also reported where the neutralizing antibodies could not be detected till 14 dpv by NPLA when pigs were vaccinated with C strain vaccine through intramuscular route, but the antibody titre increased 28 dpv and reached maximum titre at 42 dpv [14]. A drastic fall of antibody titre was observed in Group A at 180 dpv as none of the animals showed protective antibody titre. The probable reason behind not maintaining the duration of immunity up to 6 months after primary vaccination might be due to catabolism of antibodies and clearance of immunogen from host immune system. In contrast, the higher percentage of protected animal in Group B and C might be due to booster vaccination at 28 dpv. Therefore, the present study indicated that a booster vaccination is essential after 28 dpv to keep the animals protected. Vaccination with modified live CSFV also elicited increased antibody titre after booster vaccination in pigs [15]. Moreover, a sharp rise of the protective antibody titre was noticed in all the vaccinated groups at 194 dpv following revaccination at 180 dpv. In the present study, 194 dpv onwards, the percentages of animals showing protective antibody titres were 100 in all the vaccinated groups. High level of protective antibody titres in all the CSF vaccinated groups might be due to the anamnesis antibody response of booster vaccination.

In the present study (Table-2) percentage of the herd indicates the percentage of animal possessing antibody titre log_10_ 1.50 against CSFV. Further, MDA was detected in the piglets born from vaccinated sows till 42 days of age (weaning period). In the present study, the percentage of animal possessing log_10_ 1.50 MDA level declined sharply (from 83.33% to 16.67% in Group B and 100% to 66.66% in Group C) on 42 days as compared to 21 days of age. From the estimation of antibody titre in piglets at 21 and 42 days of their age, it was observed that piglets born from immune sows receiving CSF vaccine during 1 month of pregnancy had a high level of passive antibody titre in comparison to piglets born from non-immune sows. The high antibody titre in piglets of Group C might be due to higher antibody level of their mothers which was subsequently transferred to their offsprings. It is assumed that passive immunity is primarily dependent on the antibody titre of the mother and on the amount of colostrums ingested by newborn piglets [16]. It is reported that newborn piglets carried MDA at 1^st^ week and it persisted in most of them till 7^th^ weeks of their age when their mothers were vaccinated against CSFV at 90 days after conceptions [17].

## Conclusion

CSF causes major losses in pig farming, with various degrees of disease severity. Lapinised vaccines against CSFV are used routinely in endemic countries. However, despite intensive vaccination programs in endemic areas, CSF has not been eradicated. The present study was undertaken to establish an effective vaccination schedule for CSF on the basis of humoral immune responses in pigs vaccinated with cell culture adapted CSF vaccine. Based on the evaluation of humoral immune response of vaccinated pigs and MDA titre in piglets born from immunized sows, it may be concluded that the more effective vaccination schedule for prevention of the disease with cell culture adapted CSF vaccine is primary vaccination at 2 months of age followed by booster vaccination at 28 and 180 dpv and at 1 month of gestation.

## Authors’ Contributions

The present study was a part of MKN’s original research work during Ph.D. thesis program. DKS, BCD and SS had designed the plan of work. MKN and DK carried out the experiment in the farm. MKN, DKS, PD and PR carried out the laboratory work. MKN, PD, JBD and GM analyzed the results. All the authors read and approved the final manuscript.
